# Revealing the Phenolic Composition and the Antioxidant, Antimicrobial and Antiproliferative Activities of Two *Euphrasia* sp. Extracts

**DOI:** 10.3390/plants13131790

**Published:** 2024-06-28

**Authors:** Daniela Benedec, Ilioara Oniga, Daniela Hanganu, Ana-Maria Vlase, Irina Ielciu, Gianina Crișan, Nicodim Fiţ, Mihaela Niculae, Timea Bab, Emoke Pall, Laurian Vlase

**Affiliations:** 1 Department of Pharmacognosy, Faculty of Pharmacy, “Iuliu Hațieganu” University of Medicine and Pharmacy, 400010 Cluj-Napoca, Romania; dbenedec@umfcluj.ro (D.B.); ioniga@umfcluj.ro (I.O.); timea.bab@plantextrakt.ro (T.B.); 2 Department of Pharmaceutical Botany, Faculty of Pharmacy, “Iuliu Hațieganu” University of Medicine and Pharmacy, 400337 Cluj-Napoca, Romania; gheldiu.ana@umfcluj.ro (A.-M.V.); irina.ielciu@umfcluj.ro (I.I.); gcrisan@umfcluj.ro (G.C.); 3 Department of Paraclinical Sciences, Faculty of Veterinary Medicine, University of Agricultural Sciences and Veterinary Medicine Cluj-Napoca, 400372 Cluj-Napoca, Romania; nfit@usamvcluj.ro; 4 Department of Clinical Sciences, University of Agricultural Sciences and Veterinary Medicine Cluj-Napoca, 400372 Cluj-Napoca, Romania; mihaela.niculae@usamvcluj.ro (M.N.); emoke.pall@usamvcluj.ro (E.P.); 5 PlantExtrakt Ltd., 407059 Cluj-Napoca, Romania; 6 Department of Pharmaceutical Technology and Biopharmacy, Faculty of Pharmacy, “Iuliu Hațieganu” University of Medicine and Pharmacy, 400012 Cluj-Napoca, Romania; laurian.vlase@umfcluj.ro

**Keywords:** *Euphrasia officinalis* subsp. *pratensis*, *Euphrasia stricta*, polyphenols, antioxidant, antimicrobial, antiproliferative

## Abstract

The species of the genus *Euphrasia* present important medicinal potential according to their traditional uses. However, few studies aim to sustain this fact by scientific evidence. The present study aimed to explore the phytochemical profile and investigate the antioxidant, antimicrobial and antiproliferative potential of *E. officinalis* subsp. *pratensis* Fr. (EO) and *E. stricta* J.P.Wolff ex J.F.Lehm (ES). The tested samples consisted of ethanolic extracts. The identification and quantification of phenolic compounds were performed using spectrophotometric and LC–MS/MS methods. The antioxidant capacity was evaluated using the DPPH, FRAP and xanthine oxidase methods. Antimicrobial properties were screened using disk diffusion, broth microdilution and anti-biofilm assays, while antiproliferative potential was assessed on a colorectal adenocarcinoma human cancer cell line (DLD-1). The LC–MS/MS analysis showed chlorogenic acid and rutin as the dominant constituents in the tested extracts. The antioxidant activity assays showed important capacity for both samples; in vitro antimicrobial and anti-biofilm properties were exhibited, especially against Gram-positive bacteria, and an important inhibitory potential was observed on the proliferation of the DLD-1 cell line. The findings in the present study contribute to the recommendation of EO and ES for the prevention and treatment of oxidative stress-related pathologies, cancer and microbial infections.

## 1. Introduction

*Euphrasia* L. is a genus in the Orobanchaceae family, formerly included in the Scrophulariaceae family, containing semi-parasitic, herbaceous, small, annual or perennial species with purple, blue–white and violet labiate flowers or yellow spots on the lower petal [1,2,3]. It is widely spread throughout Europe, North Asia and North America and includes approximately 450 species [4,5], among which eighteen species and subspecies grow spontaneously in Romania [1]. The most well-known species of this genus are *E. officinalis* subsp. *pratensis* Fr. (formerly known as *E. rostkoviana*) [5,6,7,8,9,10,11], followed by *E. stricta* J.P.Wolff ex J.F.Lehm [3,12,13,14], *E. brevipila* Burn. and Gremli [15,16], *E. tetraqueta* (Breb.) Arrond. [17] and *E. pectinata* Ten. [18]. The species of the genus have proven to be an important source of a wide range of metabolites, such as iridoids, flavonoids, phenolic acids, lipides, essential oil and carotenoids [2,3,6,8,19,20,21,22,23].

*Euphrasia officinalis* subsp. *pratensis* Fr. (EO), commonly known as the eyebright, is widely used in traditional medicine for treating hay fever, sinusitis and other upper respiratory tract infections but, especially, as an eye lotion to treat and prevent infectious and non-infectious eye disorders (e.g., conjunctivitis, ophthalmia, ocular allergies, eye fatigue, cataract, glaucoma, corneal ulcers) [7,9,19,20,24,25]. Further pharmacological studies have also revealed the anti-inflammatory, astringent [5,9,26], anti-hyperglycemic [10], antioxidant [6,11,26], antimicrobial [2,20] and protective properties on corneal epithelial cells and photoaging of skin exposed to UVB [4,17]. Clinical studies have revealed the effectiveness and safety of using EO eye drops in inflammatory and catarrhal conjunctival diseases [25,27,28]. Moreover, EO extracts showed antiproliferative activity on different human cancerous cell lines: corneal epithelial (10,014 pRSV-T), breast (MCF-7), cervical epithelioid (HeLa), prostate (LNCaP), renal (ACHN) and amelanotic melanoma (C32) [5,18].

*Euphrasia stricta* J.P.Wolff ex J.F.Lehm (ES) is a lesser-known species of the *Euphrasia* genus, also commonly known as the eyebright and having the same traditional uses as the EO species [5,14]. It is lesser-known and studied, with most of the existing studies focusing on its antioxidant activity [12,13], attributed to its phenolic compounds [3].

In traditional Romanian medicine, only the EO species is used, in particular for the treatment of eye disorders (conjunctivitis, hordeolum, spastic photophobia, cataract, glaucoma, eye pain, ulcers, allergies, etc.) but also some respiratory and gastrointestinal diseases [6,7]. The research carried out on the species of the genus *Euphrasia* spontaneous in the Romanian flora is very limited and carried out only on EO [6,7,21].

Colorectal cancer is one of the most common malignant diseases but also one of the main causes of death in the world [29,30]. In Romania, after 2020, this type of cancer ranks first after lung and prostate cancer. In recent years, curative treatment has significantly increased the survival rate of patients; however, severe adverse effects as well as resistance to cytostatic drugs remain major disadvantages that, for some patients, limit this treatment option [30,31]. As a result, countless efforts are being made throughout the world to find alternative therapeutic solutions, especially from the plant kingdom. Polyphenolic compounds with few side-effects and low or no toxicity, due to important antioxidant, anti-inflammatory, antiangiogenic, proapoptotic and antiproliferative properties, can be considered sources of potentially beneficial molecules in the management of colorectal pathology [30,31,32].

The scarcity of studies revealing the phytochemical composition of the EO and ES species shows the necessity for more detailed phytochemical studies [3]. All this taken into consideration, the present study aimed to evaluate the phenolic compounds, one of the most important classes of secondary metabolites for the composition of these species harvested from the spontaneous Romanian flora, in order to establish possible chemical differences between them. Moreover, the present study aimed to assess important in vitro biological activities, such as the antioxidant, antimicrobial and antiproliferative activities, of the EO and ES extracts. The novelty of the present study consists in the fact that it is the first report aiming to compare two of the most frequently found species of the genus *Euphrasia* in the Romanian flora and to investigate their phytochemical composition, correlating it to their most important biological activities. Moreover, as the antiproliferative effect of these species was assessed on colorectal adenocarcinoma cell lines, the present approach appears to be the first report in the literature on the antiproliferative effect of *Euphrasia* species on colorectal adenocarcinoma cell lines.

## 2. Results

### 2.1. Spectrophotometrical Assays for the Quantification of Total Phenolic Compounds

The results obtained for the evaluation of the polyphenolic content (total polyphenols (TP) expressed as mg gallic acid equivalents (GAE)/g, total flavonoids (TF) expressed as mg rutin equivalents (RE)/g, caffeic acid derivatives (CAD) expressed as mg caffeic acid equivalents (CAE)/g and the antioxidant capacity of the two samples) are presented in Table 1. The highest amount of TP and TF was determined for the EO extract (TP: 92.10 mg GAE/g, TF: 24.72 mg RE/g), followed by the ES extract (TP: 74.91 mg GAE/g, TF: 10.81 mg RE/g). The analysis of these data (Table 1) showed significant statistical differences (*p* < 0.01) and highly significant differences (*p* < 0.001) between the two species.

### 2.2. The Antioxidant Activity

The antioxidant activity of the EO and ES ethanolic extracts was evaluated by DPPH radical bleaching and the FRAP methods. The obtained results demonstrated that both the DPPH free-radical scavenging capacity as well as the ferric ion reduction of the EO sample were higher (50.93 µg/mL and 520.21 µM TE/mL) than those of the ES sample (71.57 µg/mL and 255.33 µM TE/mL) (*p* < 0.001) but less effective than Trolox (*p* < 0.001), with these results being in relation to the total polyphenols and flavonoid values obtained after quantification.

The two studied samples showed a good xanthine oxidase inhibitory activity with higher values for ES (I% = 71.90% and I = 93.46 mg AE/mL) than EO (I% = 16.73% and I = 21.75 mg AE/mL) but lower compared to I% (90.04) for allopurinol (*p* < 0.001), proving that the ethanolic extracts obtained from the two species can provide encouraging premise for new anti-hyperuricemic natural products [33].

### 2.3. The HPLC–MS/MS Analysis

High-performance liquid chromatography coupled with mass spectrometry (HPLC–MS/MS) is a prevalent technique for the investigation of phenolic and flavonoid compounds in medicinal plant extracts. Using this method, the present study identified a total of fifteen bioactive compounds in the composition of the two ethanolic extracts (Table 2). 

The EO extract was characterized by the presence of fifteen compounds, including eight phenolic acids (protocatechuic, gentisic, caftaric, chlorogenic, vanillic, *p*-coumaric, ferulic, rosmarinic) and seven flavonoid heterosides and aglycones (catechin, hyperosid, isoquercitrin, rutin, quercitrin, luteolin, apigenin). Notably, rutin (quercetin-3-O-rutinoside, 61.57 ± 1.55 μg/mL) and chlorogenic acid (23.72 ± 0.28 μg/mL) emerged as the predominant compounds in addition to significant concentrations of *p*-coumaric acid (9.40 ± 0.20 μg/mL), rosmarinic acid (5.83 ± 0.29 μg/mL), hyperoside (6.72 ± 0.17 μg/mL) and isoquercitrin (5.59 ± 0.11 μg/mL). This analysis marked the first identification of several compounds in the composition of EO, namely gentisic acid, caftaric acid, vanillic acid, rosmarinic acid, hyperoside (quercetin-3-O-galactoside) and quercitrin (quercetin 3-O-rhamnoside). On the other hand, the ES extract revealed thirteen polyphenols, comprising both phenolic acids and flavonoids, mirroring the composition of the EO sample, although in varying quantities. Unlike EO, ES lacked vanillic acid and catechin. The analysis highlighted chlorogenic acid (353.86 ± 9.83 μg/mL), rutin (57.41 ± 1.58 μg/mL), isoquercitrin (20.38 ± 0.16 μg/mL) and *p*-coumaric acid (16.21 ± 0.25 μg/mL) as the main compounds in ES. Chlorogenic acid was detected in the ES sample at a concentration approximately 15 times higher than that in the EO sample (353.86 ± 9.83 μg/mL vs. 23.72 ± 0.28 μg/mL), indicating that ES could serve as both a significant source of chlorogenic acid, known for its notable therapeutic benefits, and a valuable marker for the chemotaxonomy of the Romanian *Euphrasia* species.

### 2.4. The Cytototoxic Activity

**Figure 1:**
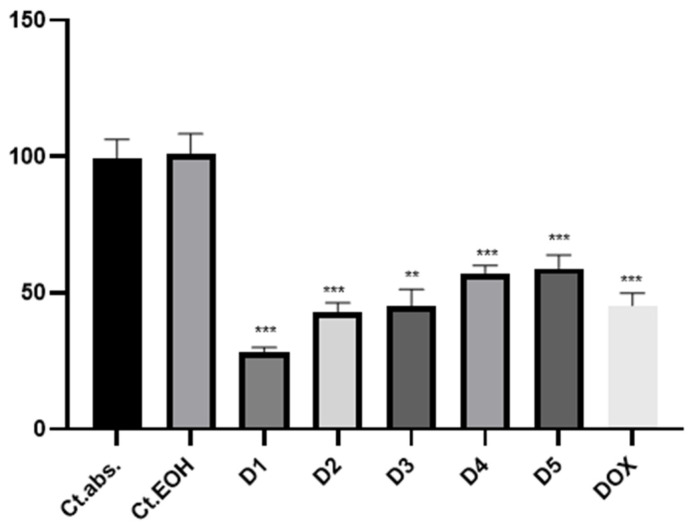
Growth inhibition of DLD cells incubated with different concentrations of ES extract (D1—1.35 μmol GAE/μL, D2—1.08 μmol GAE/μL, D3—0.81 μmol GAE/μL, D4—0.54 μmol GAE/μL, D5—0.27 μmol GAE/μL) for 24 h. Ct. abs.—absolute control, Ct.EOH—70% ethanol. The proliferation potential of the cells was assessed using the CCK-8 assay. Doxorubicin was added as a positive control, and ** *p* ≤ 0.01, *** *p* ≤ 0.001.

**Figure 2:**
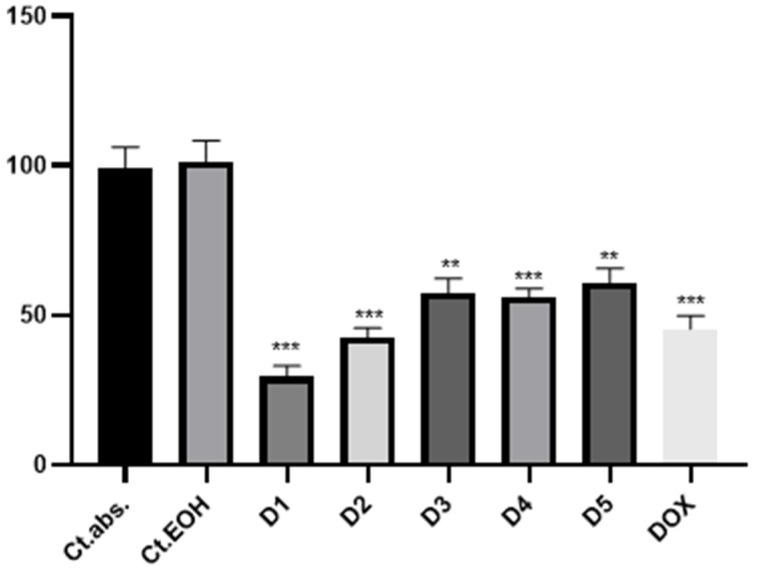
Growth inhibition of DLD cells incubated with different concentrations of EO extract (D1—1.10 μmol GAE/μL, D2—0.88 μmol GAE/μL, D3—0.66 μmol GAE/μL, D4—0.44 μmol GAE/μL, D5—0.22 μmol GAE/μL) for 24 h. Ct. abs.—absolute control, Ct.EOH—70% ethanol. The proliferation potential of the cells was assessed using the CCK-8 assay. Doxorubicin was added as a positive control, and ** *p* ≤ 0.01, *** *p* ≤ 0.001.

The viability of DLD-1 cells after 24 h of incubation with the EO and ES extracts was evaluated using the CCK-8 assay. The results of the assay indicated no toxicity for ethanol, which was the solvent used for extract preparation. The results of the antiproliferative evaluation of the ethanolic EO and ES extracts are presented in Figure 1 and Figure 2. Both extracts exhibited inhibitory potential on the proliferation of the tested DLD-1 cells after 24 h of treatment.

The viability results following exposure of DLD1 cells to the highest concentration of EO extract indicated an average viability of 29.48% ± 3.36, contrasting with the untreated culture (*p* = 0.004), reflecting a significantly lower outcome compared to the reference compound, where the average viability was 45.34% ± 4.49 (*p* = 0.001). A correlation between the extract concentration and cell viability was observed, indicating an increase in cell proliferation as the concentration of added extract decreased. More precisely, at concentration D2 after 24 h of exposure, the average cell viability was 57.47% ± 4.71 (*p* = 0.01), while at D3, a slight decrease was indicated, 55.90% ± 2.91 (*p* = 0.005). Remarkably, at the highest concentration of added extract, the average cell viability reached 60.55% ± 4.94 (*p* = 0.01). A similar trend was indicated in the results for the ES extract as well. At the highest concentration of extract added to the DLD1 cell line, the average cell viability was 28.47% ± 1.48 compared to the positive control, where the average cell viability was 45.34% ± 4.49. The results were statistically significant (*p* = 0.001). The rest of the concentrations showed a dose-dependent inhibition of cell proliferation. For D2 and D3, the average cell viability was 43.07% ± 3.21 and 45.21% ± 6.03, respectively, similar to that of the positive control, slightly higher for the concentrations D4 and D5 (57.19% ± 2.77 and 58.92% ± 4.84).

### 2.5. The Antimicrobial Activity

#### 2.5.1. The Agar-Well Diffusion Method

The in vitro antimicrobial properties of the EO and ES extracts were initially evaluated using a screening method, the agar-well diffusion method, and the obtained results are displayed in Table 3 (diameters of the inhibition zone).

Both tested samples displayed in vitro antimicrobial potential towards the microbial reference strains; still, the inhibitory activity was found mostly against Gram-positive bacteria (Table 4). The highest susceptibility was noticed in the case of *Enterococcus faecalis*, while *Pseudomonas aeruginosa* showed resistance towards both extracts. Although the EO and ES extracts presented an inhibitory effect against the proliferation of several tested organisms (MSSA—methicillin-susceptible *Staphylococcus aureus*, MRSA—methicillin-resistant *Staphylococcus aureus*, *Bacillus cereus*, *Listeria monocytogenes*, *Escherichia coli* and *Candida albicans*), their ability was significantly lower (*p* < 0.05) compared to the positive controls (gentamicin and fluconazole). The only exception was recorded against the *Enterococcus faecalis* strain, with both extracts presenting a significantly higher effect (*p* < 0.05) compared to gentamicin. Furthermore, based on the values of the diameter inhibition zones, the ES extract exhibited a superior (*p* < 0.05) antimicrobial activity compared to the EO extract against all Gram-positive bacteria.

#### 2.5.2. The Broth Microdilution Method

The MIC and MBC values established by the broth microdilution method are shown in Table 4. Similar to the results obtained using the screening method, the data indicate the EO and ES extracts possess better in vitro antimicrobial efficacy against Gram-positive bacteria. In fact, at the maximum tested concentrations, the EO and ES extracts displayed inhibitory properties towards *Escherichia coli* and *Candida albicans*. The highest antibacterial activity was recorded against MSSA, MRSA and *Bacillus cereus*. As a particular aspect, the MIC and MBC values were identical except for the ES extract that presented an MBC value two-fold higher than the MIC value against MSSA.

#### 2.5.3. The Anti-Biofilm Assay

Based on the inhibition (%) calculated values, only the ES extract presented a good anti-biofilm activity (above 50%, ++) against *Staphylococcus aureus*. Still, the same extract displayed a rather poor (0–50%, +) and no inhibition or enhancement of biofilm development and growth (<0, -) [34] against the *Listeria monocytogenes*, *Escherichia coli* and *Candida albicans* reference strains, respectively. Although the anti-biofilm activity was recorded only against *Staphylococcus aureus*, this potential was noticed for both the biofilm attachment (T0) and destruction of 24 h pre-formed biofilm (T24) (Table 5).

The anti-biofilm activity of the EO and ES extracts was described based on the inhibition (%) calculated values as good (above 50%, ++), poor (0–50%, +) and no inhibition or enhancement of biofilm development and growth (<0, -) [34].

## 3. Discussion

The two tested samples, consisting of two ethanolic extracts (70% ethanol in water *v*/*v*), are assessed for the first time for their phytochemical and biological activities. Their composition in the concentration of phenolic metabolites differs. The EO aerial part extract proved to be richer in phenolic compounds than ES, which is in agreement with other previous publications comparing the two species [3,35]. The TP amount proved to be higher for EO in comparison with a commercial preparation [17], while in a methanolic extract, the TP amount proved to be lower [11]. The standardization of the results obtained for the analysis of the Romanian EO samples was different, making a comparison with the results of the present study difficult [6]. The ES species showed lower amounts of TP compared to the results of our study for a methanolic extract in the evaluation performed by Jafri et al [12]. TF was also found in important amounts in the samples collected from Pakistan. Regarding the presence of CAD, ES showed a higher concentration (55.02 mg/g) than EO (45.08 mg/g). The only study that previously performed the quantification of these compounds was also performed on Romanian samples, but the results are difficult to compare with our results as they were expressed as rosmarinic acid equivalents [6].

The results obtained for the quantification of phenolic metabolites in the LC–MS/MS for the EO analysis align with previous research, indicating chlorogenic acid (3-O-caffeoylquinic acid) as a major component [6,19,35], while other studies have identified its isomers, 4- and 5-O-caffeoylquinic acids, in greater amounts in both EO and ES [2,3]. Caffeic acid and its derivatives (chlorogenic acid, coumaric acid) were also found to be the main class of metabolites in the Romanian EO samples, being followed by flavonoids, such as apigenin, luteolin, kaempferol, quercetin and their glycosides [6]. The same compounds were also reported for the composition of the EO infusion and 50% *v*/*v* ethanolic extracts. The present investigation reports, for the first time, the presence of caftaric and vanillic acids and confirms the presence of the other previously reported metabolites, contributing to the growing evidence on the phytochemical diversity in the *Euphrasia* genus and underscoring the therapeutic promise of these extracts.

According to several published studies, the ethanolic extract of Portuguese EO [2] showed a slightly lower antioxidant activity than our sample, and a fraction rich in glycosylated caffeic acid derivatives obtained from the methanolic extract from the species studied by Blazics et al. [6] showed strong antioxidant action. A significant antioxidant activity was also proved for the methanolic extract [11]. Regarding the ES ethanolic extract, our study is in accordance with the results on the antioxidant capacity of ES harvested from Pakistan in the DPPH and FRAP assays [12,13]. At the same time, the ethanolic and ethyl acetate EO extracts displayed strong free-radical scavenging activity, while the heptane extract did not show any essential reducing effects [9]. The extracts obtained using polar solvents (especially water and ethanol) were confirmed to showcase stronger antioxidant capacity for the DPPH and FRAP assays [2]. Additionally, the solvent not only affects the isolation of secondary metabolites but also participates in the electron and hydrogen transfer from the antioxidant to the radical, influencing the results obtained for the evaluation of the antioxidant potential. All these results were correlated with the phenolic composition of both species.

The present study brings novelty by testing, for the first time, the xanthine oxidase inhibitory activity of EO and ES samples. Our results reveal that ES has a better xanthine oxidase inhibition effect than EO, comparable to allopurinol. Phenolic compounds demonstrate inhibitory activity against xanthine oxidase (XO), an enzyme that catalyzes the oxidation of hypoxanthine to xanthine and the oxidation of xanthine to uric acid. It is involved in the production of reactive oxygen species, generating oxidative damage. The inhibition of xanthine oxidase by the different components of the extracts indicates their involvement in purine metabolism and their potential to reduce the synthesis and accumulation of uric acid at the joint level as well as to reduce the oxidative stress produced by the activity of this enzyme [33].

The results of the antimicrobial assays showed important antimicrobial potential displayed towards Gram-positive strains, both in the agar-well diffusion method and in the broth microdilution one. Similar antimicrobial potential was reported by Novy et al. [20] for both species but, for the essential oil, being attributed to its composition of palmitic acid, thymol, linalool, anethole, linolenic acid and borneol. Gram-positive strains (*Enterococcus faecalis*, *Staphylococcus aureus* and *S. epidermidis*) were also proved to be more sensitive than Gram-negative ones (*Escherichia coli, Klebsiella pneumoniae* and *Pseudomonas aeruginosa*). Similar to our results, *P. aeruginosa* was the only organism that was not inhibited by the tested samples [20]. Other types of samples tested for antibacterial potential were EO infusions and 50% *v*/*v* ethanolic extracts, proving a similar trend towards Gram-positive bacteria (*S. aureus*, *S. epidermidis*, *Micrococcus luteus*) [2,5]. This higher potential towards Gram-positive bacteria was also reported for EO extracts [2] and *Euphrasia brevipila* aerial parts petroleum ether, chloroform and ethylacetate extracts, with the biological effect depending on the solvent polarity [36]. Silver nanoparticles (AgNPs) containing an EO leaf aqueous extract were found active against *Pseudomonas aeruginosa* KACC 14021, *Escherichia coli* CCARM 0237, *Vibrio parahaemolyticus* KACC 15069 and *Staphylococcus aureus* KCTC 3881 based on the inhibition zone diameter values (15.3 mm, 11.7 mm, 14.7 mm and 13.7 mm, respectively). These AgNPs also inhibited *S. aureus* and *P. aeruginosa* biofilm formation [37]. The novelty of the present study consists of reporting the antimicrobial activity of EO and ES ethanolic extracts on MSSA and MRSA strains but also on *L. monocytogenes*. The anti-biofilm activity of these samples was also evaluated, to the best of our knowledge, for the first time in the present study. The results obtained in the present study for testing the bioactivities of the two species of the *Euphrasia* genus bring important information on the potential of the species in confirming the traditional uses of these species for the treatment of eye disorders, such as conjunctivitis and blepharitis, that can be frequently associated with bacterial infections.

The antiproliferative activity was evaluated for other species of the *Euphrasia* genus, such as *E. pectinata*, but its effects were assessed on human breast cancer, human epithelioid cervix carcinoma, hormone-dependent prostate carcinoma, renal cell adenocarcinoma and amelanotic melanoma [38]. The protective activity of EO was assessed to exist on UVB-exposed human corneal cells [17], on UVB-irradiated photoaging in normal human dermal fibroblasts [4] and on human corneal epithelial cells [9]. The results showed that the treatment with EO extracts did not cause alterations to the cell viability but it significantly prevented cell apoptosis following UVB irradiation [4]. Ethanolic and ethyl acetate EO extracts are the samples that proved the lowest toxicity to human corneal cells [9]. Concerning the antiproliferative activity, the present study brings novelty by testing, for the first time, the antiproliferative activity in vitro on colorectal adenocarcinoma cell lines. In this way, important premises for testing future bioactivities of the two species are offered. Results can be connected with the ones obtained for the antioxidant and even antimicrobial assays, as all of them may be related to the phenolic composition of these samples.

## 4. Materials and Methods

### 4.1. Plant Material

The plant materials were represented by the aerial parts collected from the spontaneous flora of Romania during the flowering period from two medicinal species of Euphrasia: *E. officinalis* subsp. *pratensis* Fr.—EO (Valea Ariesului, Romania) and *E. stricta* J.P.Wolff ex J.F.Lehm—ES (Rimetea village: 46°27′14″ N, 23°34′02″ E, Alba County, Romania). The two samples (EO and ES) with voucher allocation numbers 126.18.4.1 (EO) and 126.18.8.1 (ES) were identified at the Department of Pharmaceutical Botany of the “Iuliu Hațieganu” University of Medicine and Pharmacy Cluj-Napoca, Romania.

### 4.2. Chemical Agents

The high-purity chemical reagents used in all analyses were purchased from several companies distributing chemical substances manufactured by MercK KGaA, Darmstadt, Germany (Arnow’s and Folin-Ciocâlteu reagents, ethanol, methanol, aluminum chloride, sodium acetate, hydrochloric acid, DPPH = 2, 2-diphenyl-1-picrylhydrazyl, acetonitrile, ammonium acetate and acetic acid are all of HPLC grade); Alfa Aesar USA-Germany (iron trichloride, sodium hydroxide, sodium carbonate); Sigma-Aldrich, St. Louis, MO, USA (quercitrin, isoquercitrin, hyperoside, rutin, apigenin, quercetin, myricetin, fisetin, kaempferol, and chlorogenic, caffeic, *p*-coumaric acids); Carl-Roth, Karlsruhe, Germany (patuletin, luteolin and ferulic, gentisic, gallic, sinapic, rosmarinic acids); Dalton PharmaToronto, Canada (caftaric and cichoric acids); Sigma-Aldrich Chemie GmbH Steinheim, Germany (protocatechuic, vanillic, syringic acids, epicatechin, catechin, Trolox = 6-hydroxy-2,5,7,8-tetramethylchroman-2-carboxylic acid), TPTZ = 2,4,6-Tris(2-pyridyl)-s-triazine). The spectrophotometer that measured the absorbance was an Agilent Cary 60 UV–vis (Inc. Headquarters, Santa Clara, CA, USA).

### 4.3. Extraction Method

Air-dried aerial parts of EO and ES were grounded using a Grindomix GM 200 knife mill (Éragny, France). The 10% ethanolic extracts (70% ethanol in water *v*/*v*) were obtained from the air-dried aerial parts of the EO and ES species, grounded and extracted with 70%. The 10% ethanolic extracts (70% ethanol in water *v*/*v*) were obtained from air-dried aerial parts of EO and ES species, ground and extracted with 70% ethanol. Thus, 10 g of plant material were extracted with 100 mL of 70% ethanol for 30 min at 60 °C on a water bath under a bulb condenser. They were then filtered into a volumetric flask and made up to 100 mL with 70% ethanol. The extracts were subsequently filtered through paper filters and then centrifuged (at 4500 rpm, 10 min). The supernatant solutions were collected and used for analysis [39,40].

### 4.4. Spectrophotometrical Assays

The total polyphenolic (TP), total flavonoid (TF) and caffeic acid derivative (CAD) contents in the two ethanolic extracts were spectrophotometrically determined according to the methods in the pharmacopoeia [Romanian Pharmacopoeia, European Pharmacopoeia] with specific reagents (Folin–Ciocâlteu, AlCl_3_ and Arnow reagents), and the results were expressed as equivalents of gallic acid (mg GAE)/g dried plant product), rutin (mg RE/g dried plant product) and caffeic acid (mg CAE/g dried plant product).

For the determination of the TP content, 2 mL of sample diluted with ethanol was mixed with 1 mL of Folin–Ciocalteu reagent, 10 mL of distilled water and sodium carbonate (29%) to 25 mL. The sample was incubated in the dark for 30 min, and the absorbance was measured at 760 nm. The content of total polyphenols was expressed as mg of gallic acid equivalents extracted from 1 mL extract or 1 g of dried plant material, and the values were calculated using a calibration curve plotted with five concentrations of gallic acid (R^2^ = 0.999).

For determination of the TF content, 5 mL of each extract was mixed with 5 mL of sodium acetate (10%) and 3 mL of aluminum chloride (25%) and filled up to 25 mL with methanol in a calibrated flask. The absorbance was measured at 430 nm, and the TF content value was expressed as rutin equivalent (RE) using a calibration curve plotted with five concentrations of rutin (R^2^ = 0.992). The results are expressed as mg of rutin equivalents extracted from 1 mL extract or 1 g of dried plant material.

For the determination of the CAD content, 1 mL of each extract was mixed with 1 mL of hydrochloric acid (0.5 N), 1 mL of Arnows’ reagent (10 g sodium nitrite and 10 g sodium molybdate in 100 mL distilled water), 1 mL sodium hydroxide and water in a calibrated flask (10 mL). The absorbance was measured at 500 nm, and the content value was calculated using a calibration curve plotted with five concentrations of caffeic acid (R^2^ = 0.989). The values of CAD contents were expressed as mg caffeic acid equivalents extracted from 1 mL extract or 1 g of dried plant material [39,41,42,43].

### 4.5. LC–MS/MS Analysis of Phenolic Compounds in Euphrasia Extracts

LC–MS/MS analysis is a critical tool for characterizing phenolic compounds in vegetal extracts due to its high sensitivity, specificity and accuracy. This study aimed to utilize a rapid LC–MS/MS methodology for the analysis of *Euphrasia* sp. extracts. The analysis was conducted using an Agilent 1100 HPLC Series system consisting of a degasser, column thermostat, binary gradient pump, autosampler and UV detector coupled with an Agilent 1100 Series LC/MSD Trap system mass spectrometer, following previously validated methods [44,45]. For the analysis of epicatechin, catechin and the acids syringic, gallic, protocatechuic and vanillic, a different LC–MS method previously outlined by Rusu was employed, with adjustments in the binary gradient for component separation. Quantitative analysis was performed with Agilent ChemStation software B01.03 and DataAnalysis version 5.3, allowing for the identification of phenolic compounds based on MS/MS spectra and retention times, compared against standards. Quantification was achieved through peak area evaluation against calibration curves of standards, with results presented in µg compound/mL extract, across a concentration range of 0.5–50 µL/mL, achieving a correlation coefficient (R^2^) greater than 0.999 [46]. Additionally, rosmarinic acid (RA) quantification employed a specific LC–MS/MS method previously published by our research group [39,45,47], using the same HPLC system but with a mobile phase of acetonitrile and 1 mM ammonium acetate in water. RA was identified based on its retention time of 2.2 min and its MS/MS spectral data. The assay was conducted using standard solutions ranging from 40 to 640 ng/mL, achieving a correlation coefficient (R^2^) of 0.999.

### 4.6. Antioxidant Activity

The antioxidant capacity of the EO and ES extracts was examined by the DPPH radical scavenging method and the FRAP test (ferric reducing antioxidant power) [42,43]. Evaluation of the antioxidant efficiency was also performed by quantifying the decrease in the xanthine oxidase activity [33].

#### 4.6.1. DPPH Assay

A total of 2 mL of EO/ES extract at different concentrations was added to 2 mL of a DPPH methanolic solution at a concentration of 0.1 g/L and maintained at 40 °C in a thermostatic bath for 30 min. Changes in absorbance were measured at 517 nm. The percentage of inhibition of DPPH·was calculated according to the following formula: Inhibition (I%) = [(Ac − As)/Ac] × 100, where Ac = absorbance of negative control and As = absorbance of sample or of the Trolox solution after 40 min [39,48,49,50]. IC_50_, the half maximal inhibitory concentration, measuring the potence of metabolites from the tested samples to inhibit the activity of reactive oxygen species, was used to quantify the DPPH·inhibition. The assays were performed in triplicate [42,43,51,52].

#### 4.6.2. FRAP Assay

The FRAP results for the assay that assessed the reduction of iron from ferric ion to ferrous ion by the 2,4,6-tripyridyl-s-triazine (TPTZ) radical were expressed as mM Trolox equivalents/mL extract using a calibration curve (R^2^ = 0.989) constructed with 10–40 mg/L Trolox standard. The FRAP reagent consisted of a mixture of 2.5 mL of a 10 mM TPTZ solution in 40 mM HCl mixed with 2.5 mL 20 mM ferric chloride solution and 25 mL of acetate buffer at a pH of 3.6. Four mL of the EO/ES extract was diluted to 1.8 mL with water and mixed with 6 mL FRAP reagent. The blank solution was prepared using water instead of the sample. The absorbance was measured at 450 nm. Trolox was used as a reference, and a calibration curve was plotted (R^2^ = 0.994). The results were expressed as µM Trolox equivalents/100 mL extract. The assays were performed in triplicate [39,48,49,50].

#### 4.6.3. Xanthine Oxidase Assay

The xanthine oxidase inhibitory activity of the two *Euphrasia* extracts was evaluated in vitro spectrophotometrically using xanthine as a substrate for the enzyme and allopurinol as a positive control. The following solutions were used: 0.5 U/mL xanthine oxidase solution, 0.15 mM xanthine solution and phosphate buffer (pH 7.4) and ultrapurified water. Samples: to each 1.5 mL sample containing 1.5 µL extract (ES, respectively ER) diluted in ultrapurified water, 3.9 mL phosphate buffer and 0.3 mL xanthine oxidase (0.5 U/mL) were added. Then, after incubation for 10 min at 25 °C, 4.5 mL xanthine was added and incubated for another 30 min at 25 °C. Control: 3.9 mL phosphate buffer and 0.3 mL xanthine oxidase were diluted with 1.5 mL ultrapurified water. The mixture was incubated for 10 min. at 25 °C, and then, 4.5 mL xanthine was added and incubated for another 30 min at the same temperature. Allopurinol (0.3 mg/mL) was used as the standard (positive control). The negative control (the mixture of 9 mL ultrapurified water, 3.9 mL phosphate buffer and 0.3 mL xanthine oxidase) was incubated for 40 min at 25 °C. Allopurinol in a different concentration was used for the calibration curve (R^2^ = 0.987). Absorbance was read at 293 nm using a UV–vis spectrophotometer (Techcomp UV2500, Livingston, UK, double beam) [33].

### 4.7. Cytotoxicity Assays

The antiproliferative potential of EO and ES extracts against the colorectal adenocarcinoma cell line DLD-1 (CCL-221™, purchased from the American Type Culture Collection and provided by Dr. Eva Fisher-Fodor and Dr. Olga Șoritau at the Oncological Institute “Prof. dr. Ion Chiricuță” from Cluj-Napoca) was investigated using the CCK8 assay. DLD-1 cells were cultured in RPMI-1640 medium (Gibco Life Technologies, Paisley, UK) supplemented with 10% fetal bovine serum (Sigma-Aldrich, St. Louis, MO, USA), 1% glutamine (Sigma-Aldrich, St. Louis, MO, USA) and 1% antibiotics–antimycotics (Gibco Life Technologies, Paisley, UK). The cultures were maintained at 37 °C with 5% CO_2_ and 60% humidity. DLD-1 cells (1 × 10^4^ cells/well) were exposed to each sample at five different concentrations (0.27 μmol GAE, 0.54 μmol GAE, 0.81 μmol GAE, 1.08 μmol GAE and 1.35 μmol GAE for EO and 0.22 μmol GAE, 0.44 μmol GAE, 0.66 μmol GAE, 0.88 μmol GAE and 1.10 μmol GAE for ES). These concentrations were calculated based on the TP concentration expressed in μmol GAE/μL. The positive control consisted of doxorubicin (reference compound) at a concentration of 20 μg/mL, while the negative control comprised cells maintained in standard culture medium. The potential inhibitory effect was also evaluated for the solvent used for preparing hydroalcoholic products. The extracts were incubated for a further 24 h. Following 24 h of incubation, CCK-8 solution (Sigma-Aldrich, St. Louis, MO, USA) was added to each well, and the cell cultures were further incubated for 4 h at 37 °C in the dark. CCK-8 contains a water-soluble tetrazolium salt that is reduced by viable cells to produce a colored formazan dye. The amount of formazan dye produced is directly proportional to the number of viable cells in the sample [53,54,55]. Subsequently, the absorbance of each well was measured at 450 nm using a microplate reader (Bio-Rad, Hercules, CA, USA). All experiments were performed in triplicate and expressed as mean  ±  SD. The calculation of cell survival (%) was performed based on the optical densities correlated with the optical density of the control.

### 4.8. Antimicrobial Activity Assays

#### 4.8.1. Agar-Well Diffusion Method

The EO and ES extracts were investigated in terms of in vitro antibacterial and antifungal activity using an agar-well diffusion method [56] according to EUCAST (European Committee on Antimicrobial Susceptibility Testing) criteria [57]. The evaluation was performed against the following reference strains: *Staphylococcus aureus* ATCC 25923 (methicillin-susceptible *S. aureus*, MSSA), *Staphylococcus aureus* ATCC 700699 (methicillin-resistant *S. aureus*, MRSA), *Bacillus cereus* ATCC 14579, *Enterococcus faecalis* ATCC 29219, *Escherichia coli* ATCC 25922, *Pseudomonas aeruginosa* ATCC 27853 and *Candida albicans* DSMZ 1386. The microbial reference strains were purchased from Oxoid Ltd. (Hampshire, UK). Positive and negative controls were evaluated as well, namely two standard antimicrobial disks: gentamicin (10 µg) and fluconazole (25 µg) (Oxoid Ltd., Hampshire, UK) and 70% ethanol in water *v*/*v*, respectively. Following a 24-h incubation, pure microbial strains were added to sterile distilled water and prepared as turbidity 0.5 McFarland (1.0 × 10^6^ CFU/mL) standard (bio-Meriuex, Marcy l’Etoile, France) equivalent inoculum. Each freshly made inoculum was placed onto specific agar plates: Mueller–Hinton (MH) and Sabouraud dextrose (SD) (Merck, Darmstadt, Germany), MH and SD for bacteria and *C. albicans*, respectively. From the inoculated agar plates, 6-mm diameter wells were cut to allow for the addition in three wells of 50 μL for each tested product (extracts, negative control). These plates were incubated at 37 °C for 24 h for bacteria and 48 h for *C. albicans*. The diameters of the growth inhibition zone were measured, with their corresponding values recorded in mm. This in vitro evaluation was performed in duplicate [51,58].

#### 4.8.2. Broth Microdilution Method

The EO and ES extracts were further evaluated employing the broth microdilution method [51] that allows for the establishment of parameters such as the minimum inhibitory (MIC), bactericidal (MBC) and fungicidal (MFC) concentrations. The tested products were prepared as two-fold serial dilutions using 100 µL of specific broth (MH and SD, respectively) and sterile flat-bottomed 96-well microtiter plates (Deltalab, Barcelona, Spain). Each dilution was inoculated with a volume of 5.0 µL microorganism inoculum and incubated at 37 °C for 24 h for bacteria and 48 h for *C. albicans*; then, the wells were visually examined against the controls (the two types of broths used to culture bacterial and fungal species (MH and SD, respectively)). The absence of turbidity was indicative of in vitro inhibitory activity, and its corresponding highest dilution was recorded as the MIC value. After the MIC value reading, a volume of 10.0 µL was sampled from each well to be inoculated on specific agar plates and cultured for 24 h and 48 h for bacteria and *C. albicans*. No colony growth pointed out the MBC and MFC values. As for the agar well diffusion method, positive and negative controls were added, namely gentamicin 50 mg/mL (Sigma-Aldrich, St. Louis, MO, USA), fluconazole (10–1000 μM) (Sigma-Aldrich, St. Louis, MO, USA) and 70% ethanol in water *v*/*v*, and the testing was performed in duplicates for each tested extract.

#### 4.8.3. Anti-Biofilm Assay

The antimicrobial activity of the EO and ES extracts was also tested against the biofilm formation considering two stages, namely biofilm attachment (T0) and 24 h pre-formed biofilm (T24), based on previously reported protocols [34,38,59]. For T0, microbial inoculums were made using the same technique as the agar-well diffusion method. Sterile flat-bottomed 96-well microtiter plates were inoculated with equal volumes (100 μL) of each *Euphrasia* extract and microbial inoculum and incubated without shaking for 24 h at 37 °C. For T24, the plates were inoculated with each microbial inoculum and incubated for 24 h to allow for biofilm formation and further exposed to equal volumes of each extract. With the same testing conditions, the controls, represented by organisms + specific broth, bacteria + MH broth + gentamicin and *C. albicans* + SD broth + Fluconazole, were evaluated. After 24 h incubation, the crystal violet staining (CVS) assay was employed to quantify the biofilm biomass.

For each well of the plate, the content was removed, followed by plate washing (three times using sterile distilled water). After drying, the adhered cells were fixed using 96% methanol (150 μL) and further stained with 0.1% crystal violet solution (100 μL) (Sigma-Aldrich, St. Louis, MO, USA). After 20 min at room temperature, these plates were repeatedly washed with sterile distilled water and further treated with 150 μL of 100% ethanol. Following gentle shaking, the optic density (OD) was read at 490 nm using a microplate reader Sunrise™ (Tecan, Männedorf, Switzerland) with the results expressed as percentage inhibition based on the following equation: Inhibition (%) = (OD_control_ − OD_extract)_/OD_control_ × 100 [34,51]. The in vitro anti-biofilm activity of each extract was described based on these calculated values (%) as good (above 50%, ++), poor (0–50%, +) and no inhibition or enhancement of biofilm development and growth (<0, -) [34].

### 4.9. Statistical Data Analysis

All the above-described methods were employed in duplicate or triplicate with the results presented as mean ± standard. The statistical analysis was performed by one-way analysis of variance (ANOVA) with the following values: *p* < 0.05 threshold value for statistical significance, *p* < 0.001 very significant, 0.001  <  *p*  <  0.05 significant and *p*  >  0.05 insignificant.

## 5. Conclusions

The present study scientifically substantiates the therapeutic uses of two species belonging to the genus *Euphrasia* by capitalizing on the polyphenolic content in direct connection with the antimicrobial, antioxidant, antiproliferative and hypouricemic effects. In fact, the mechanism behind the antiproliferative and antimicrobial effects is related to the reduction in oxidative stress. Specifically, phenolic acids (caffeic acids and their derivatives) followed by flavonoids (apigenin, kaempferol and quercetin) were found to be the main metabolites in the composition of the two species. Antioxidant effects were related to the inhibition of xanthine oxidase activity. Antimicrobial effects were proved, especially against Gram-positive bacteria, and antiproliferative effects were proved on colorectal adenocarcinoma cell lines. There are limitations related to the results obtained that are related to the discrepancies between in vitro results and physio-pathological processes, which may result in challenges in translating these results in clinical applications. Obviously, future preclinical investigations are needed and correlations with in vivo studies are also necessary. However, these preliminary results offer an important basis and prove the potential use of *E. officinalis* ssp. *pratensis* and *E. stricta* in the treatment of disorders caused by oxidative stress and microbial infections as well as in gout.

## Figures and Tables

**Table 1:** Results obtained for the quantification of polyphenolic contents and antioxidant activity of EO and ES extracts.

Sample	TP (mg GAE/g)	TF (mg RE/g)	CAD (mg CAE/g)	DPPH (IC_50_ µg/mL)	FRAP (µM TE/mL)	XO	
						I%	I (mg AE/mL)
EO	92.10 ± 2.90	24.72 ± 0.29	45.08 ± 1.92 ^c^	50.93 ± 2.19 ^d^	520.21 ± 13.79	16.73 ±0.35 ^f^	21.75 ± 0.51 ^g^
ES	74.91 ± 1.28 ^a^	10.81 ± 0.19 ^b^	55.02 ± 1.87	71.57 ± 3.42 ^d^	255.33 ± 9.67 ^e^	71.90 ± 1.38 ^f^	93.46± 0.42
Trolox	-	-		11.88 ± 0.02		-	-
Allopurinol	-	-	-	-	-	90.04 ± 2.35	-

Note: GAE (gallic acid equivalents), RE (rutin equivalents), CAE (caffeic acid equivalents), TE (trolox equivalents), AE (allopurinol equivalents). Each value is the mean ± SD of three independent measurements. Lowercase letters in the same row indicate significant differences: ^a^ *p* < 0.01 (EO vs. ES); ^b^ *p* < 0.001 (EO vs. ES); ^c^ *p* < 0.01 (ES vs. EO); ^d^ *p* < 0.01 (trolox vs. EO, ES); ^e^ *p* < 0.001 (EO vs. ES); ^f^ *p* < 0.01 (allopurinol vs. EO, ES); ^g^ *p* < 0.001 (ES vs. EO).

**Table 2:** Results obtained for the quantification of phenolic compounds by LC–MS/MS for the EO and ES extracts.

No.	Phenolic Compound	*m*/*z* Value	tR ± SD (min)	Concentration (μg/mL) EO	Concentration (μg/mL) ES
1.	Protocatechuic acid	153	2.80 ± 0.01	1.85 ± 0.05	0.87 ± 0.03
2.	Gentisic acid	179	3.52 ± 0.04	0.46 ± 0.02	<0.02
3.	Caftaric acid	311	3.54 ± 0.05	0.31 ± 0.02	<0.02
4.	Chlorogenic acid	353	5.62 ± 0.05	23.72 ± 0.28	353.86 ± 9.83
5.	Vanillic acid	167	6.70 ± 0.01	1.14 ± 0.01	-
6.	*p*-Coumaric acid	163	9.48 ± 0.08	9.40 ± 0.20	16.21 ± 0.25
7.	Ferulic acid	193	12.8 ± 0.10	2.32 ± 0.08	4.80 ± 0.01
8.	Catechin	289	6.00 ± 0.07	0.26 ± 0.01	-
9.	Rosmarinic acid	359	2.20 ± 0.02	5.83 ± 0.29	0.82 ± 0.03
10.	Hyperoside	463	19.00 ± 0.2	6.72 ± 0.17	1.336 ± 0.01
11.	Isoquercitrin	463	19.90 ± 0.10	5.59 ± 0.11	20.38 ± 0.16
12.	Rutin	609	20.20 ± 0.15	61.57 ± 1.55	57.41 ± 1.58
13.	Quercitrin	447	23.64 ± 0.13	<0.02	6.90 ± 0.15
14.	Luteolin	285	29.10 ± 0.19	0.46 ± 0.02	0.53 ± 0.02
15.	Apigenin	269	33.10 ± 0.15	0.67 ± 0.03	1.06 ± 0.02

**Table 3:** Results obtained for the quantification of the in vitro antimicrobial activity of the EO and ES extracts using the agar-well diffusion method.

Tested Products	Diameters of Inhibition Zone (mm)							
	MSSA	MRSA	*Bacillus* *cereus*	*Enterococcus faecalis*	*Listeria monocytogenes*	*Escherichia coli*	*Pseudomonas* *aeruginosa*	*Candida albicans*
EO	14.50 ± 0.50 ^a^	12.00 ± 0.71 ^a^	13.75 ± 0.43 ^a^	12.5 ± 0.50	12.75 ± 0.43 ^a^	9.75 ± 0.43 ^a^	0	10 ± 0.00 ^c^
ES	17.00 ± 0.71 ^a^	16.25 ± 0.43 ^b^	15 ± 0.00 ^a^	14 ± 0.00	14.75 ± 0.43 ^a^	9.75 ± 0.43 ^a^	0	10 ± 0.00 ^c^
Gentamicin	19 ± 0.00	17 ± 0.25	20 ± 0.00	10 ± 0.00 ^a^	22 ± 0.50	19 ± 0.00	18 ± 0.00	-
Fluconazole	-	-	-	-	-	-	-	21 ± 0.00

MSSA—methicillin-susceptible *Staphylococcus aureus*, MRSA—methicillin-resistant *Staphylococcus aureus*. Values represent means of duplicate determinations (*n* = 2) ± standard deviations. Lowercase letters in the same column point out significant differences: ^a^ *p* < 0.05 (extract vs. gentamicin); ^b^ *p* > 0.05 (extract vs. gentamicin); ^c^ *p* < 0.05 (extract vs. fluconazole). Gentamicin (10 μg/disk) and fluconazole (25 µg) were included as positive controls.

**Table 4:** Results obtained for the quantification of the in vitro antibacterial activity of the EO and ES extracts using the broth microdilution assay.

Samples	Microorganisms													
	MSSA		MRSA		*Bacillus* *cereus*		*Enterococcus* *faecalis*		*Listeria* *monocytogenes*		*Escherichia* *coli*		*Candida* *albicans*	
	MIC	MBC	MIC	MBC	MIC	MBC	MIC	MBC	MIC	MBC	MIC	MBC	MIC	MFC
EO	27.08	27.08	27.08	27.08	27.08	27.08	54.17	54.17	54.17	54.17	54.17	>54.17	54.17	>54.17
ES	5.50	22.02	11.01	11.01	11.01	11.01	44.05	44.05	44.05	44.05	44.05	>44.05	44.05	>44.05
Gentamicin MIC (mg/L)	3		4		3		3		3		4		-	
Fluconazole MIC (mg/L)	-		-		-		-		-		-		8	

“-“ = not active; MIC: minimum inhibitory concentration (μmol GAE/mL)/; MBC: minimum bactericidal concentration (μmol GAE/mL); MFC: minimum fungicidal concentration (μmol GAE/mL).

**Table 5:** The results obtained for the assessment of the anti-biofilm activity of the EO and ES extracts.

			% Inhibition					
Samples	*Staphylococcus aureus*		*Listeria monocytogenes*		*Escherichia* *coli*		*Candida* *albicans*	
	T0	T24	T0	T24	T0	T24	T0	T24
EO	+	+	+	-	-	-	-	+
ES	++	++	+	+	-	-	-	+
Gentamicin	+	++	+	++	-	++	-	-
Fluconazole	-	-	-	-	-	-	++	++

## Data Availability

The original contributions presented in this study are included in the article; further inquiries can be directed to the corresponding author/s.
